# Prophylactic Administration of Doxycycline Reduces Central Venous Catheter Infections in Patients Undergoing Hematopoietic Cell Transplantation

**DOI:** 10.4084/MJHID.2013.015

**Published:** 2013-02-16

**Authors:** Mohamed Baydoun, Zaher K. Otrock, Samar Okaily, Rita Nehme, Racha Abu-Chahine, Ali Hamdan, Samar Noureddine, Souha Kanj, Zeina Kanafani, Ali Bazarbachi, Mohamed A. Kharfan-Dabaja

**Affiliations:** 1 Division of Hematology-Oncology and Bone Marrow Transplantation Program, American University of Beirut Medical Center, Beirut, Lebanon; 2 School of Nursing, American University of Beirut Medical Center, Beirut, Lebanon; 3 Division of Infectious Diseases, American University of Beirut Medical Center, Beirut, Lebanon

## Abstract

Hematopoietic stem cells are generally transfused through a central venous catheter (CVC), which also facilitates administration of medications and intravenous fluids. We had observed a high rate of CVC infections at our Bone Marrow Transplantation (BMT) unit. Accordingly, we evaluated the impact of administration of doxycycline as a prophylactic strategy to reduce CVC infection rates. Data was collected retrospectively on 54 consecutive patients, 26 who received doxycycline (doxycycline group), and we compared their outcomes to a previous cohort of 28 subjects who did not receive doxycycline (comparison group). The groups were comparable in regards to age, gender, transplant type, and CD34 cell dose. No (0%) CVC infection was observed in the doxycycline group, while 5 infection episodes (18%) occurred in 4 patients in the comparison group (p<0.001). Isolated organisms included: Escherichia-coli (EC)=1, coagulase-negative Staphylococcus-spp (CNSS)=2, both EC & CNSS=1. Notwithstanding the non-randomized comparative nature of our study, results suggest that CVC infection rate was reduced significantly after adding doxycycline for prophylaxis. A randomized controlled study is warranted to confirm these findings.

## Introduction

Hematopoietic cell transplantation (HCT) is an important and perhaps the only known curative treatment modality for patients with hematologic malignancies and non-malignant hematologic disorders. Patients usually receive high-dose of chemotherapy followed by infusion of hematopoietic stem cells either from him/herself or from a related or unrelated donor. As a result, patients might experience undesirable side effects including nausea, vomiting, anorexia, diarrhea, and oral mucositis.^1^–^3^ Moreover, patients’ white blood cell counts decrease significantly resulting in impairment of immunity, hence predisposing HCT recipients to infections which might be fatal. Accordingly, prevention of infections is of vital importance in these cases.^3^–^4^

Hematopoietic stem cells are usually infused through a CVC. At our center, CVCs are inserted upon admission and before the administration of conditioning chemotherapy. Catheter-related bloodstream infections (CR-BSI) are also a recognized and potentially serious complication associated with CVC devices. This is because a CVC can be a vulnerable host for microorganisms to colonize and multiply inducing blood-stream infections.^5^ Prevention of infections associated with CVC is an utmost priority due to its adverse impact on transplant outcomes. The detrimental economic impact of hospital acquired infections transcend to the society at large by potentially resulting in a prohibitive increase in health care costs. Hence, the importance of prevention is emphasized.

Doxycycline, a relatively inexpensive antimicrobial, prescribed at a dose of 100 mg orally twice-daily administered on the day of stem cell infusion, was started on all adult HCT recipients since January 2011 in an attempt to decrease the unusually high rate of CVC-related infections observed at the Bone Marrow Transplantation (BMT) unit of the American University of Beirut Medical Center (AUBMC). We conducted this study to assess the efficacy of doxycycline in reducing CVC infections and compared outcomes against a historic control.

## Patients and Methods

This study was approved by the Institutional Review Board of the American University of Beirut. Data was collected retrospectively from the medical records on consecutive HCT patients transplanted between October 2009 and October 2011, who received (doxycycline group) or did not receive doxycycline (comparison group). Patients in the comparison group were transplanted between October 07, 2009 and January 21, 2011; whereas subjects in the doxycycline group underwent their transplantation from January 26, 2011 to October 20, 2011. The selected time period included an almost equal number of patients in both groups. Only adult patients (age ≥ 18 years) were eligible for inclusion in this comparative analysis. Collected variables included demographic and patients’ characteristic data including age, gender, diagnoses, type of transplant (allogeneic or autologous), and preparative chemotherapy regimens used. In addition, information on CVC site and infection status, isolated microorganisms, and clinical outcomes were also reviewed.

CVCs, mostly inserted via the subclavian vein, were always implanted by a general or vascular surgeon at bedside using local anesthesia and following aseptic techniques. The same caliber antiseptic catheter (Arrow International, Reading, PA, USA) was used, double-lumen for autologous HCT and triple-lumen for allogeneic HCT throughout the study period. All patients received high-dose chemotherapy as part of conditioning, hematopoietic stem cells, and therapeutic support consisting of fluids, medications and/or transfusion of blood products. Irrespective of the doxycycline use to prevent CVC infections, all patients received fluoroquinolone prophylaxis consisting of levofloxacin 500 mg orally daily during severe neutropenia (defined as an absolute neutrophil count (ANC) <500 cells/μL), later replaced by a third generation cephalosporin, namely cefepime or equivalent, if fever in the setting of neutropenia ensued. The CVC was kept in place for an average of 19 ± 3 days prior to its removal. The CVC tip was always be sent for culture according to our standard institutional practice.

Criteria for the diagnosis of CVC infection were defined as follows: (1) a clinical infection with positive blood cultures from the catheter with or without positive peripheral blood cultures; (2) a positive catheter tip culture after removal of the catheter for suspicion of a catheter-related infection. Adult patients who underwent HCT and developed fever within 48 hours after CVC insertion were excluded. This was considered in order to avoid including subjects who could have potentially acquired infection during CVC insertion.

### Statistical analysis

Data were analyzed using the Statistical Package for the Social Sciences software version 18.0 (SPSS, Chicago, IL, USA). The continuous study variables were described by their median and range, whereas categorical variables were described by their relative frequencies and counts. Groups were compared for their demographic and clinical variables using chi-square and student t tests. Differences were considered statistically significant if a p value was less than 0.05.

## Results

Data was collected retrospectively on 54 consecutive patients, 26 in the doxycycline group, and 28 in the comparison group. The median age of patients in the doxycycline and comparison groups were 46 (20–75) years and 41 (23–65) years, respectively (p=0.325). Groups were also similar for recipient gender (p=0.132), HCT type (p=0.234), and number of CD34+ cells infused at transplantation (p=0.223) (Table 1).

### Neutrophil engraftment kinetics

Addition of doxycycline did not affect time-to-neutrophil engraftment (13 days vs. 12 days, p=0.164).

### Infection rates and associated findings

No (0%) CVC infection occurred in the doxycycline group, while 5 (18%) infection episodes were recorded in 4 patients in the comparison group, resulting in a statistically significant difference (p < 0.001). Characteristics of patients who developed CVC are summarized in Table 2. Patients developed high-grade fever requiring a comprehensive work-up including peripheral and central blood culture testing. Subjects had marked leukopenia (and neutropenia) at the time of blood culture testing (ANC <500 cells/μL) in all cases. The median time from CVC insertion to blood sampling for culture testing was 13.5 (10–21) days. Isolated organisms included *Escherichia coli* in one patient, coagulase-negative *Staphylococcus spp* (CNSS) in 2 patients, and both *Escherichia coli* and CNSS were recovered from the same patient from 2 catheter lumens. Antimicrobial susceptibility testing showed that 3 isolates were resistant to tetracycline, 1 was susceptible, and susceptibility was not available in 1 case. Interestingly, the CVC tip cultures for the above-mentioned 4 patients were negative for any bacterial growth.

## Discussion

CVCs play an essential role in facilitating essential care to immunocompromised patients requiring treatments such as chemotherapy, blood products, parenteral nutrition, antibiotics and antimicrobial agents. Despite improvements in line care, CVC associated infections remain a serious complication in HCT recipients, particularly during the pre-engraftment phase of the procedure.^6^–^7^ These infections result in increased morbidity and even death among HCT recipients.^8^–^9^ Catheter infection generally arises from bacterial colonization of the internal surface of the intravascular catheter. Microorganisms causing CVC infections gain access to the bloodstream through either the skin at the catheter insertion site or through the catheter hub.^5^,^10^

In HCT patients, all CVCs were required to be inserted following the highest sterile barrier precautions standards .^11^–^12^ The preferred approach to minimize infections is implementation of the central line-associated blood-stream infection (CLABSI) bundle, which consists of hand hygiene, full barrier precautions, cleaning the insertion site with chlorhexidine, avoiding femoral sites for insertion and removing unnecessary catheters.^13^ Studies have demonstrated that implementation of CVC care bundle has been associated with a significant reduction in CLABSI.^14^ Although the efficacy of the CLABSI prevention bundle has not been studied in HCT recipients, all five elements of the bundle are commonly recommended for this particular population. As a matter of fact, CLABSI prevention bundle was implemented on all patients (doxycycline and comparison groups) throughout the specified study period.

Other measures to decrease the risk of CVC infections have been studied. Antibiotic- impregnated catheters specifically with minocycline/rifampin have been shown to decrease CLABSI in HCT patients requiring subclavian central venous access.^15^–^16^ In one retrospective study, minocycline/rifampin-impregnated catheters did not affect the susceptibility of staphylococci to tetracyclines or rifampin.^15^ A meta-analysis conducted by Ramritu et al.^17^ for the relative effectiveness of antibiotic-coated CVC, based on five randomized clinical trials in intensive care units, showed a significant reduction in catheter-related blood-stream showed infection associated with the use of these catheters compared to uncoated catheters.^17^

We had observed an unusually high rate of CVC-related infections at the BMT unit of the AUBMC. As a result, we implemented evidence-based care to ameliorate the situation including abiding by the hand hygiene policy, maximal barrier precautions, proper handling and manipulating of CVC, minimizing handling and maintaining a closed system, limiting manipulation to certified and competent staff, and ensuring aseptic technique during CVC insertion procedures. Moreover, we had already initiated the CLABSI prevention bundle, but without achieving a lower CVC infection rate. The possibility of using antibiotic-coated catheters was entertained. However, this option was not found to be feasible as it would have resulted in a prohibitive increase in the cost of transplantation in Lebanon. We opted instead to initiate all our adult HCT recipients, whether receiving an autologous or an allogeneic HCT, on prophylactic doxycycline at a dose of 100 mg orally twice per day; doxycycline is a broad spectrum antibiotic that is relatively inexpensive and generally well tolerated. Doxycycline has been started at our transplant unit since January 2011 for all adult BMT patients starting on the day of transplant till the day of removing the CVC. Notwithstanding incorporation of doxycycline, we did not change our routine practice of using fluoroquinolone prophylaxis during neutropenia. It is important to emphasize that hematologic and immunologic reconstitution differs between recipients of autografts and allografts; and that administration of antibacterial prophylaxis is not universally applied, especially during autologous HCT.

Interestingly, 3 of the 4 subjects who developed CVC related infections had received autologous HCT. The median (in days) length of hospital stay was similar between subjects in the doxycycline and comparison groups (22 (14–78) days vs. 22 (14–42) days, p=0.403), respectively.

One might argue that the use of a broad-spectrum antibiotic could potentially increase the risk of bacterial resistance which represents a serious problem from the medical standpoint. While this argument holds true, one should also consider the serious morbidities and high risk of mortality, resulting from CVC infections in HCT recipients; and their adverse consequences in regards to potential for prolonged hospitalization and consequently increase in cost of health care, among others. Accordingly, it seems obvious that the adverse risks resulting from a serious CVC infection are likely to outweigh the possibility of developing drug resistance by using doxycycline prophylaxis. It is important to keep in mind that bacterial isolates might be resistant to doxycycline as in our case.

Our results are noteworthy, as no CVC infections were observed in the doxycycline group compared to five infections in the historic control group. To our knowledge, there are no published studies that specifically evaluate the use of doxycycline for prevention of CVC infections in the HCT setting. The small size of the groups as well as the small number of events did not allow us to determine the relationship between incidences of CVC infections among various diseases. Nevertheless, this question ought to be addressed in a larger study. Other limitations of our study include the retrospective nature, heterogeneity in regards to diagnoses and type of transplant performed, and conditioning regimens used. The results reported herein shall provide the scientific basis to develop a large randomized multicenter controlled trial to confirm our results.

## Figures and Tables

**Table 1:** Patient and treatment characteristics

Variables	Doxycycline- treated group (n=26)	Comparison group (n=28)	p-value
Median age (range), years	46 (20–75)	41 (23–65)	0.325
Gender	Male = 13 Female = 13	Male = 21 Female = 7	0.132
Type of HCT	Autologous=21 Allogeneic=5	Autologous=22 Allogeneic=6	0.234
Median CD34^+^ cells (range), ×10^6^ cells/kg	8.40 (3.71–12.6)	6.30 (3.28–13.4)	0.223

HCT . Hematopoietic Stem Cell Transplantation

**Table 2:** Characteristics of subjects who developed CVC infections

Age/gender	Disease	Type of transplantation	CVC type	WBC ×10^6^/L*	Presence of fever (temperature in °C)	Time from catheter insertion to blood culture (days)	Blood culture growth results
36/M	AML	Allogeneic	TL	800	38.3	21	coagulase-negative Staphylococcus spp.
26/F	HL	Autologous	DL	200	38.9	13	Escherichia coli
64/M	NHL	Autologous	DL	100	39.8	14	coagulase-negative Staphylococcus spp. and Escherichia coli
61/M	MM	Autologous	DL	200	38.7	10	coagulase-negative Staphylococcus spp.

Abbreviations: M: male; F: female; AML: acute myelogenous leukemia; HL: Hodgkin lymphoma; NHL: non-Hodgkin lymphoma; MM: multiple myeloma; TL: triple-lumen; DL: double-lumen.

* at time of diagnosing the CVC-related infection.
